# Experiences of In-Group and Out-Group Skin Tone Discrimination and Their Associations with Incident Cardiovascular Disease Among African American Adults in the Jackson Heart Study

**DOI:** 10.1007/s40615-025-02590-8

**Published:** 2025-08-07

**Authors:** Sydney A. Barlow, Jessica R. Fernandez, Juliana S. Sherchan, Ellis P. Monk, Jaime Slaughter-Acey, Mario Sims, Allana T. Forde

**Affiliations:** 1 Division of Intramural Research, National Institute on Minority Health and Health Disparities, National Institutes of Health, Bethesda, MD USA; 2 Department of Sociology, Harvard University, Cambridge, MA USA; 3 Department of Epidemiology, Gillings School of Global Public Health, University of North Carolina at Chapel Hill, Chapel Hill, NC USA; 4 Department of Social Medicine, Population and Public Health, University of California, Riverside School of Medicine, Riverside, CA USA

**Keywords:** Cardiovascular disease, Jackson heart study, Optimism, Skin tone discrimination

## Abstract

**Background:**

African American adults face an elevated risk of cardiovascular disease (CVD) compared to other racial and/or ethnic groups in the USA. Although discrimination has been linked to this disparity, the relationship between skin tone discrimination and CVD incidence remains scarce. This study investigated the associations of in-group discrimination (from African American individuals) and out-group skin tone discrimination (from White individuals) with incident CVD and whether these associations differed by sex and optimism.

**Methods:**

This study analyzed data from 3519 African American participants (aged 21–95 years) in the Jackson Heart Study from 2000 to 2016. Cox Proportional Hazards regression assessed associations between skin tone discrimination and CVD (including stroke and coronary heart disease (CHD)). Each CVD component, along with heart failure (HF), was also analyzed separately. Models adjusting for sociodemographic characteristics, health behaviors, and CVD risk factors estimated hazard ratios (HR) and 95% confidence intervals (CI). Interaction terms were included in the fully adjusted models to assess the moderating roles of sex and optimism.

**Results:**

Over the 16-year follow-up, 8.0% of participants developed CVD, 3.9% developed stroke, 4.9% developed CHD, and 7.3% developed HF. Participants who reported that Black individuals treated them better than other Black individuals because of their skin tone had an increased risk of CVD (HR 1.33, 95% CI 0.95–1.83). Out-group skin tone discrimination, whether better or worse treatment, was associated with a higher incidence of CHD (HRs ranged from 1.23 to 1.43), although CIs were wide. These associations did not vary by sex. Optimism moderated the association between out-group skin tone discrimination and HF, such that those who reported worse treatment and had the highest level of optimism had the greatest risk of HF. Optimism did not moderate the associations between in-group skin tone discrimination and the CVD outcomes.

**Conclusions:**

These findings highlight the differential impact of in-group and out-group skin tone discrimination on cardiovascular health. Better in-group treatment was marginally linked to a higher CVD risk, while out-group skin tone discrimination, whether better or worse treatment, marginally increased CHD risk. Skin tone discrimination may therefore be a unique risk factor for CVD for African American individuals.

**Supplementary Information:**

The online version contains supplementary material available at 10.1007/s40615-025-02590-8.

## Introduction

Profound cardiovascular health disparities disproportionately impact African American adults in the USA. Systemic racism has been identified as one of the main driving forces behind the growing racial disparities in cardiovascular disease (CVD) [1, 2]. Systemic racism is a form of racism that is deeply embedded in systems, laws, and practices and includes the interpersonal discrimination that many people of color face [3]. Interpersonal discrimination, defined as an individual’s implicit or explicit racial bias influencing their interactions and view of others [4], extends to day-to-day interactions in which people are treated unfairly because of their race or (other factors) and is a biopsychosocial stressor that can impact health [5]. According to the biopsychosocial model of racism, discrimination affects health through physiological responses to stress that activate the hypothalamic–pituitary–adrenal (HPA) axis leading to the release of the stress hormone, cortisol. There is evidence to suggest that discrimination as a stressor contributes to adverse cardiovascular health outcomes [6, 7].

Skin tone discrimination, a specific form of discrimination, may adversely affect cardiovascular health. Skin tone discrimination is a type of prejudice or discrimination that occurs when people favor individuals with a lighter skin complexion over a darker skin complexion and can exist in two forms: inside one’s ethnic group (in-group discrimination) and outside of one’s ethnic group (out-group discrimination) [8, 9]. The USA has a long history of skin tone discrimination dating back to the enslavement of African people in the USA. During the 1800s, enslaved people with lighter skin engaged in forced labor in the “Slave Master’s house,” while enslaved people with darker skin were forced to perform strenuous manual labor in the field [8, 10, 11]. However, being forced to work inside the house did not lessen the overall degrading experience of the enslavement of Black people. These skin tone preferences continue to exist, where individuals with lighter compared to darker skin benefit from certain advantages, including higher socioeconomic status, increased perceived attractiveness, and reduced experiences of discrimination from Black and non-Black racial groups[12, 13]. Given the deep historical footprint of skin tone discrimination in the USA, skin tone discrimination may uniquely play an important role in the cardiovascular health of African American individuals.

Research suggests that skin tone discrimination may be particularly harmful to the health of African American individuals. Having darker skin was associated with greater cardiometabolic risk[14], allostatic load[15], and cumulative biological risk[16]. Several studies [14–17] used skin tone (i.e., “light” “dark”) as a proxy for general skin tone discrimination, whereas other studies [10, 17, 18] directly measured the frequency of perceived discrimination based on skin tone and delved into differences between in-group and out-group discrimination. In-group skin tone discrimination was associated with higher incidence and severity of cardiovascular and cardiometabolic disorders[18] and poor self-rated physical health[10] among African American individuals. African American individuals may face a double burden of racism-related stress stemming from experiences of discrimination related to race and skin tone.

The association between skin tone discrimination and CVD among African American adults may differ by sex, but evidence has been mixed. One study found no sex differences in the relationship between skin color discrimination (from both White and African American individuals) and cardiovascular and cardiometabolic health[18]. Whereas another study of African American adults revealed that the relationship between skin color and cumulative biological risk varied by sex, discrimination did not explain the health differences for women. Women with darker skin had greater physiological deterioration compared to their counterparts with lighter skin, and this association was not significant for men[16].

In addition to sex, protective and adaptive factors have been associated with the cardiovascular health of African American individuals[19]. One particular factor is optimism, defined as the tendency to expect favorable outcomes[20]. Prior studies suggested that optimism was associated with ideal cardiovascular health[20–22] for Black and non-Black racial groups. In addition to the direct association between optimism and cardiovascular health, evidence suggested that optimism moderated the relationship between perceived discrimination and diastolic blood pressure reactivity[23], healthcare utilization[24], and health behaviors[25]. However, one study found that high levels of optimism were associated with a poor immune system[26], and others found no association with optimism and overall or cause-specific mortality[27] or diet or alcohol use [28]. As such, optimism may buffer or exacerbate the impact of skin tone discrimination on CVD.

Although there is a growing body of research on skin tone discrimination, studies focusing on the impact of skin tone discrimination on cardiovascular disease are sparse. In addition, most of these studies did not examine or distinguish between in-group and out-group skin tone discrimination. Additionally, most of these studies used a cross-sectional study design, which limited the ability to make causal inferences. No studies, to our knowledge, examined the association of skin tone discrimination with CVD using a longitudinal study design. Furthermore, prior research on sex differences [16, 18] in the relationship between skin tone discrimination and cardiovascular outcomes is limited and yielded inconsistent findings. Although prior research examined optimism as a moderator of the association between discrimination and health outcomes, this relationship has yet to be examined in the context of skin tone discrimination and incident CVD.

As an extension of the existing literature on skin tone discrimination, the current study (1) assessed the associations of skin tone discrimination (in-group, out-group) with incident CVD, (2) examined if these associations varied by sex, and (3) explored whether levels of optimism moderated this association.

## Methods

### Data Source

The Jackson Heart Study (JHS) is a large, prospective cohort study that was designed to investigate the social, environmental, and biological factors contributing to CVD in African American adults[29]. Participants (aged 21–95 years) in the JHS were recruited from three counties (Hinds, Madison, Rankin) in the Mississippi metropolitan area. Participants were enrolled in the JHS from four sources: a random sample from the Accudata America commercial listing (17%), volunteers (30%), those already participating in the Atherosclerosis Risk in Communities (ARIC) study (31%), and secondary family members of existing JHS participants (22%)[30, 31]. In total, 5306 participants were recruited at visit 1 (2000–2004) and completed two additional follow-up visits: visit 2 (2005–2008, 79.2% of participants from baseline retained) and visit 3 (2009–2013, 90.8% of visit 2 participants retained) [32]. Demographic characteristics, medical history, and genetic information were collected from self-reported questionnaires, in-clinic examinations, and home interviews. Annual follow-up interviews were administered via telephone[31]. Additional details about the JHS are described elsewhere [31, 32].

### Study Population

This study used data from visits 1–3 and the second annual follow-up questionnaire. Of the 5306 participants initially recruited at visit 1, those with missing data on stroke, coronary heart disease (CHD), or heart failure (HF) (*n* = 1211), skin tone discrimination at visit 1 (*n* = 186), or optimism from the second annual follow-up questionnaire (*n* = 280) were excluded from the analysis. Participants were also excluded if they were missing data on age, sex, education, occupation, alcohol intake, smoking status, physical activity, body mass index (BMI), hypertension status, and diabetes status (*n* = 200). Two “missing” categories were created for income (*n* = 523) and diet (*n* = 286) to retain a larger sample size for the analyses. The final analytic sample was 3519 after applying the exclusion criteria. The JHS study was reviewed and approved by the Institutional Review Boards at the University of Mississippi Medical Center, Jackson State University, and Tougaloo College. All participants provided written informed consent.

### Cardiovascular Health Outcomes

CVD incidence was assessed using CVD event data. The individual cardiovascular outcomes were also analyzed separately.

#### Cardiovascular Disease

Incident CVD was a variable developed using pre-existing stroke and coronary heart disease (CHD) event data from 2000 to 2016[33]. Heart failure was not included in the CVD definition because heart failure data collection began in 2005. Trained professionals monitored and surveilled CVD events. Surveillance procedures consisted of (1) an annual follow-up where trained professionals contacted participants by telephone and (2) medical record abstraction, which included retrieving death certificates and discharge lists from state departments or hospitals. Additional details are outlined elsewhere[34]. Incident CVD was categorized as (1) developed stroke or CHD over the follow-up period and (0) did not develop stroke or CHD over the follow-up period.

#### Stroke

Stroke events were based on information collected from hospitalizations and reported deaths occurring inside and outside the hospital. Stroke events were characterized as definite or probable stroke according to the National Survey of Stroke[33]. A definite or probable stroke was defined as “a sudden or rapid onset of neurological symptoms lasting for > 24 hours or leading to death” [33–35] or absent otherwise. Additional details are described elsewhere[33]. Incident stroke was categorized as (1) stroke diagnosis over the follow-up period and (0) no stroke diagnosis.

#### Coronary Heart Disease

CHD was based on hospitalization (non-fatal) or death (fatal) from a myocardial infarction (MI) and cardiac procedures. This information was obtained from medical history, death certificates, and other hospital documents[33]. Incident CHD events were defined as definite or probable hospitalized myocardial infarction, probable or definite fatal CHD, or the occurrence of a cardiac procedure. Additional details are described elsewhere[34]. Incident CHD was categorized as (1) CHD diagnosis over the follow-up period and (0) no CHD diagnosis.

#### Heart Failure

HF was based on hospital inpatient and outpatient HF criteria[33, 34]. The HF classifications included (1) definite decompensated HF, (2) probable decompensated HF, (3) chronic stable HF, (4) unlikely HF, and (5) unclassifiable. HF diagnosis was present if an event was categorized as (1) “Chronic Stable HF” or “Definite or Probable Decompensated HF” and (0) otherwise. Incident HF was categorized as (1) HF diagnosis over the follow-up period and (0) no HF diagnosis.

### Skin Tone Discrimination

JHS participants were asked two questions about the degree of skin tone discrimination they encountered (relative to other Black individuals) from Black individuals (i.e., in-group skin tone discrimination) and White individuals (i.e., out-group skin tone discrimination) captured at visits 1 and 3. Out-group skin tone discrimination was assessed using the following question: “Because of the shade of your skin color, do you think White people treat you a lot better, somewhat better, no different, somewhat worse, or a lot worse than other Blacks.” In-group skin tone discrimination was assessed using the following question: “Because of the shade of your skin color, do you think Black people treat you a lot better, somewhat better, no different, somewhat worse, or a lot worse than other Blacks.” Responses for both questions were categorized as (0) no different, (1) worse (“somewhat worse” or “a lot worse”), and (2) better (“somewhat better” and “a lot better”) treatment.

### Moderators

Self-reported sex (male or female) and optimism were examined as moderators. Optimism, obtained from the second annual follow-up questionnaire, was assessed using 6 items from the Life Orientation Test-Revised (LOT-R). Participants were asked to rate how much they related to each of the three “positively worded” statements and each of the three “negatively worded” statements: Three items were positively worded: (1) “In uncertain times I expect the best, (2) I’m always optimistic about my future, (3) Overall, I expect more good things to happen than bad.” The remaining three items were “negatively worded”: (1) “If something can go wrong for me, it will, (2) I hardly ever expect things to go my way, (3) I rarely count on good things happening to me.” Responses to each of the six items included (1) “not at all like me,” (2) “somewhat like me,” (3) “a little like me,” and (4) “a lot like me,” and “negatively worded” items were reversed coded. Optimism was categorized into tertiles (i.e., low, medium, and high) to identify threshold effects[22].

### Covariates

Models were adjusted for age (continuous), sex (male, female), educational attainment, income, occupation, smoking status, alcohol intake, physical activity, diet, BMI, diabetes status, and hypertension status, which could be potential confounding variables or mediators. Educational attainment was based on the highest level of education: (1) less than high school diploma, (2) high school graduate/general equivalency diploma, and (3) vocational school, trade school, or college graduate. Income was based on family income, family size, and poverty level and categorized as: (1) poor (< poverty level), (2) lower-middle (1–1.5 times the poverty level), (3) upper-middle (> 1.5 to < 3.5 times the poverty level), (4) affluent (≥ 3.5 times the poverty level), and (5) missing. Occupation was based on the classifications from the standard occupational manual developed by the US Department of Labor and included (1) managerial/professional, (2) service, (3) sales, (4) construction, (5) production, and (6) other (farming, military, sick, unemployed, retired, student). Smoking status was categorized as (0) never, (1) former, and (2) current. Alcohol intake was measured as the average number of drinks in the past 12 months. Physical activity was categorized as (0) poor health, (1) intermediate health, and (2) ideal health. Poor health, based on the American Heart Association (AHA) definition, was defined as 0 min of moderate and vigorous physical activity; intermediate health was < 150 min of moderate activity, or < 75 min of vigorous physical activity, or < 150 min of combined moderate and vigorous physical activity. Ideal health was defined as  ≥ 150 min of moderate activity, or ≥ 75 min of vigorous activity, or ≥ 150 min of combined moderate and vigorous physical activity. Similarly, diet was categorized as (0) poor health, (1) intermediate health, (2) ideal health, and (3) missing. Diet (per the American Heart Association) was based on the components that comprise a 2000 kcal diet. The components included fruits and vegetables, ≥ 4.5 cups/day; fish, > 3.5 oz, twice per week; sodium, < 1500 mg/day; sugary beverages, < 450 kcal/week; and whole grains, ≥ 3 servings/day. Poor health was defined as 0 to 1 components included in the diet, intermediate health included 2 to 3 components, and ideal health included 4 to 5 components in the diet. BMI was included as a continuous measure of participant weight divided by height squared (kg/m^2^).

Hypertension status was based on the average of two measurements of systolic and diastolic blood pressure (BP) and self-reported BP medication status. Participants with a hypertension status of “yes” had a blood pressure ≥ 140/90 mmHg, as defined by the Seventh Report of the Joint National Committee (JNC7), or used any blood pressure lowering medication, and “no” otherwise. Diabetes status was based on measures of fasting plasma glucose and hemoglobin A1c (HbA1c), defined by the American Diabetes Association (ADA 2010), and self-reported diabetic insulin medication status. Participants with a diabetes status of “yes” had a fasting glucose ≥ 126 mg/dL, HbA1c ≥ 6.5%, or used diabetic medication within 2 weeks prior to visit 1 and “no” otherwise.

### Statistical Analysis Plan

Analyses were performed using Statistical Analysis Software (SAS version 9.4; SAS Institute Inc, Cary NC). Baseline characteristics were presented using means and standard deviations for continuous variables and frequencies with percentages for categorical variables. Differences in the distribution of sociodemographic characteristics at visit 1 across levels of skin tone discrimination were assessed using chi-square tests for categorical data and analysis of variance (ANOVA) tests for continuous data. Cox proportional hazards regression estimated hazard ratios (HR) and 95% confidence intervals (CI) for unadjusted and adjusted associations. Participants who did not develop CVD over the follow-up period were censored at their last visit.

For each CVD outcome (CVD overall, stroke, CHD, HF), a series of regression models were created to examine the relationship between the outcome and each type of skin tone discrimination (in-group, out-group). Model 1 was unadjusted; model 2 adjusted for age and sex; model 3 included model 2 variables and socioeconomic factors (educational attainment, income, and occupation); model 4 included model 3 variables and health behaviors (smoking status, alcohol intake, physical activity, diet, and BMI); and model 5 included model 4 variables and CVD risk factors (hypertension and diabetes).

Next, an interaction term for skin tone discrimination and sex was included in the fully adjusted model (model 5) to assess whether sex moderated the associations between in-group and out-group skin tone discrimination and each CVD outcome. In-group and out-group skin tone discrimination were both analyzed separately, and an interaction term was included in the fully adjusted model for each outcome, totaling eight analyses. The same process was conducted for optimism as a moderator. Statistical significance at a *p*-value < 0.10 represented statistically significant interaction.

The abovementioned analyses were repeated in sensitivity analyses, which captured changes in discrimination status from visit 1 to visit 3 (i.e., time varying discrimination) since discrimination is context and time-dependent [36–39], as well as may change in nature, importance, and intensity[40].

## Results

Table 1 includes visit 1 population characteristics across levels of skin tone discrimination. On average, participants were 53.5 years of age; most were female (65%), attended vocational/trade school/college (66%), never smoked (71%), had a poor diet (63%), and did not have diabetes (80%).

**Table 1:** Characteristics of Jackson Heart Study participants in the total population and by skin tone discrimination

Demographic characteristics	Total	In-group skin tone discrimination				Out-group skin tone discrimination			
	*N* = 3,519	No difference *n* = 2,574	Worse *n* = 502	Better *n* = 443	*p*-value	No difference *n* = 2165	Worse *n* = 479	Better *n *= 875	*p*-value
Age, mean ± SD*	53.5 ± 12.3	53.3 ± 12.3	53.4 ± 12.4	54.5 ± 12.6	0.15	53.4 ± 12.5	51.4 ± 11.5	54.7 ± 12.3	< 0.001
Sex, %					0.12				< 0.001
Female	64.9	64.4	68.9	63.7		66.7	55.5	65.7	
Male	35.1	35.6	31.1	36.3		33.3	44.5	34.3	
Education, %					< 0.001				0.06
< High school degree	14.5	12.9	17.5	20.5		14.6	11.1	16.3	
High school degree/GED^*^	19.3	18.3	20.9	23.3		18.8	19.2	20.6	
Vocational/trade school/college	66.2	68.8	61.6	56.2		66.7	69.7	63.1	
Income, %					< 0.001				0.006
Poor	10.7	9.7	13.5	13.3		10.3	10.9	11.4	
Lower, middle	19.2	18.3	20.3	23.5		17.6	21.5	22.2	
Upper, middle	26.5	26.3	25.9	28.4		26.1	24.6	28.7	
Affluent	28.7	30.6	25.1	21.7		30.3	28.6	24.7	
Missing	14.9	15.1	15.1	13.1		15.7	14.4	13.0	
Occupation, %					0.006				0.52
Managerial/professional	38.5	40.5	33.1	33.6		39.4	38.6	36.3	
Service	23.2	21.7	25.7	29.1		22.3	22.1	26.1	
Sales	18.5	18.6	19.3	16.7		19.0	17.1	18.1	
Construction	5.5	5.4	5.8	5.6		5.3	6.7	5.3	
Production	13.5	13.0	15.7	13.8		13.2	14.8	13.6	
Other	0.8	0.8	0.4	1.1		0.8	0.6	0.7	
Alcohol, mean ± SD	1.5 ± 5.3	1.5 ± 5.2	1.6 ± 5.5	1.5 ± 5.5	0.95	1.4 ± 5.0	2.0 ± 5.8	1.7 ± 5.7	0.04
Smoking status, %					0.09				0.92
Never	71.3	71.3	67.9	75.4		71.4	70.1	71.9	
Former	11.4	11.3	13.9	8.8		11.1	12.5	11.3	
Current	17.3	17.4	18.1	15.8		17.5	17.3	16.8	
Physical activity, %					0.95				0.88
Poor health^‡^	46.2	46.5	45.2	45.6		46.8	45.7	45.0	
Intermediate health	33.3	32.9	34.3	34.5		33.1	32.8	34.3	
Ideal health	20.5	20.5	20.5	19.9		20.1	21.5	20.7	
Diet^§^, %					< 0.001				0.98
Poor health	63.2	65.2	55.4	60.3		63.3	64.5	62.1	
Intermediate health	27.4	26.0	32.7	29.6		27.2	26.5	28.3	
Ideal health	1.3	1.1	1.4	2.3		1.2	1.3	1.5	
Missing	8.1	7.7	10.6	7.9		8.2	7.7	8.1	
Body mass index (kg/m^2^),mean ± SD	31.8 ± 7.2	31.7 ± 7.0	32.2 ± 7.3	31.9 ± 7.7	0.34	31.8 ± 6.9	32.1 ± 7.8	31.7 ± 7.4	0.53
Hypertension status^∥^, %					0.54				0.78
No	48.1	47.8	50.4	47.4		48.3	49.1	47.2	
Yes	51.9	52.2	49.6	52.6		51.7	50.9	52.8	
Diabetes status^#^, %					0.82				0.69
No	79.8	80.1	79.7	78.8		80.2	80.0	78.9	
Yes	20.2	19.9	20.3	21.2		19.8	20.0	21.1	

^*^Abbreviations: *SD*, standard deviation; *GED*, general equivalency diploma

^†^*p*-values based on chi-square (X2) and analysis of variance (ANOVA) tests

^‡^Physical activity: poor health (0 min of moderate or vigorous physical activity), intermediate health (moderate < 150 min and vigorous < 75 min), ideal health (moderate ≥ 150 min and vigorous ≥ 75 min)

^§^Diet: poor health (0–1 components); intermediate health (2–3 components); ideal health (4–5 components)

∥Hypertension status: yes (blood pressure greater than 140/90 mmHg or use of any blood pressure lowering medication)

^#^Diabetes status: yes (fasting glucose ≥ 126 mg/dL or HbA1c ≥ 6.5%; or use of diabetic medication within 2 weeks prior to visit 1; no (fasting glucose less than 126 mg/dL, a glycated hemoglobin < 6.5%; and no use of diabetes medications)

Regarding the in-group skin tone discrimination item, most participants reported no difference in treatment based on skin tone (73%), followed by worse treatment (14%) and better treatment (12%). There were significant group differences across the categories of in-group skin tone discrimination for education, income, occupation, and diet (Table 1). Regarding the out-group skin tone discrimination item, most participants reported no difference in treatment based on skin tone (62%), followed by better treatment (25%) and worse treatment (14%). There were significant group differences across the categories of out-group skin tone discrimination for age, sex, income, and alcohol intake (Table 1).

Over the follow-up period, there were 282 incident cases of CVD, 136 incident cases of stroke, 172 incident cases of CHD, and 258 incident cases of HF. Proportions and person-years of follow-up for each CVD outcome are provided in Table 2.

**Table 2:** Proportions of cardiovascular disease among Jackson Heart Study participants by categories of discrimination

		Total				Female				Male			
		N	N of incident cases	Proportion of incidence	Total Person-years of follow-up	N	N of incident cases	Proportion of incidence	Total person-years of follow-up	N	N of incident cases	Proportion of incidence	Total person-years of follow-up
Cardiovascular disease													
In-group discrimination	No difference	2574	201	0.08	31,893.41	1657	116	0.07	21,637.10	917	85	0.09	11,256.31
	Worse	502	34	0.07	6407.75	346	22	0.06	4467.75	156	12	0.08	1940.00
	Better	443	47	0.11	5645.97	282	29	0.10	3599.37	161	18	0.11	2046.60
Out-group discrimination	No difference	2165	162	0.07	27,625.76	1444	97	0.07	18,823.24	721	65	0.09	8802.52
	Worse	479	36	0.08	6180.79	266	15	0.06	3510.66	213	21	0.10	2670.14
	Better	875	84	0.10	11,140.57	575	55	0.10	7370.32	300	29	0.10	3770.26
Stroke													
In-group discrimination	No difference	2574	96	0.04	33,446.08	1657	55	0.03	21,911.51	917	41	0.04	11,534.57
	Worse	502	16	0.03	6483.15	346	13	0.04	4502.04	156	3	0.02	1981.11
	Better	443	24	0.05	5751.75	282	15	0.05	3687.21	161	9	0.06	2064.54
Out-group discrimination	No difference	2165	85	0.03	28,028.31	1444	51	0.04	19,034.13	721	34	0.05	8994.18
	Worse	479	13	0.03	6295.22	266	5	0.02	3559.16	213	8	0.04	2736.06
	Better	875	38	0.04	11,357.43	575	27	0.05	7507.47	300	11	0.04	3849.97
Coronary heart disease													
In-group discrimination	No difference	2574	125	0.05	33,289.43	1,657	72	0.04	21,861.89	917	53	0.06	11,427.55
	Worse	502	20	0.04	6473.79	346	10	0.03	4523.45	156	10	0.06	1950.34
	Better	443	27	0.06	5789.89	282	17	0.06	3695.92	161	10	0.06	2093.97
Out-group discrimination	No difference	2165	92	0.04	27,997.45	1,444	57	0.04	19,039.56	721	35	0.05	8957.89
	Worse	479	29	0.06	6243.89	266	12	0.05	3540.02	213	17	0.08	2703.86
	Better	875	51	0.06	11,311.76	575	30	0.05	7501.66	300	21	0.07	3810.10
Heart failure													
In-group discrimination	No difference	2574	183	0.07	27,608.39	1,657	116	0.07	17,917.42	917	67	0.07	9690.97
	Worse	502	34	0.07	5327.59	346	22	0.06	3705.47	156	12	0.08	1622.12
	Better	443	41	0.09	4717.91	282	23	0.08	3008.00	161	18	0.11	1709.92
Out-group discrimination	No difference	2165	159	0.07	23,152.73	1,444	101	0.07	15,592.48	721	58	0.08	7560.25
	Worse	479	32	0.07	5172.64	266	17	0.06	2887.20	213	15	0.07	2285.44
	Better	875	67	0.08	9328.53	575	43	0.07	6151.21	300	24	0.08	3177.32

### Cardiovascular Disease

In-group skin tone discrimination was marginally associated with a higher incidence of overall CVD. Participants who reported that Black individuals treated them better than other Black individuals due to their skin tone had a 33% higher risk of developing CVD overall compared to those who reported no difference in treatment based on skin tone, although confidence intervals were wide (HR 1.33 [95% CI 0.95–1.83]) (Table 3, model 5). No significant differences in overall CVD risk were observed for those reporting worse treatment compared to the no difference group (HR 0.87 [95% CI 0.59–1.24]) (Table 3, model 5).

**Table 3:** Associations between skin tone discrimination and incident cardiovascular disease

		HR (95% CI)				
		Model 1^†^	Model 2^‡^	Model 3^§^	Model 4^||^	Model 5^#^
Cardiovascular disease (CVD)*						
In-group discrimination						
No difference		1.00	1.00	1.00	1.00	1.00
Better		1.36 (0.98, 1.85)	1.28 (0.92, 1.74)	1.24 (0.89, 1.70)	1.27 (0.91, 1.74)	1.33 (0.95, 1.83)
Worse		0.87 (0.59, 1.23)	0.87 (0.59, 1.23)	0.85 (0.58, 1.21)	0.84 (0.57, 1.19)	0.87 (0.59, 1.24)
Out-group discrimination						
No difference		1.00	1.00	1.00	1.00	1.00
Better		1.29 (0.99, 1.67)	1.18 (0.90, 1.53)	1.14 (0.87, 1.48)	1.16 (0.89, 1.51)	1.16 (0.88, 1.51)
Worse		0.99 (0.68, 1.41)	1.10 (0.75, 1.56)	1.05 (0.72, 1.49)	1.03 (0.70, 1.47)	1.02 (0.69, 1.45)
Stroke						
In-group discrimination						
No difference		1.00	1.00	1.00	1.00	1.00
Better		1.45 (0.90, 2.22)	1.35 (0.85, 2.08)	1.31 (0.81, 2.02)	1.37 (0.85, 2.13)	1.41 (0.87, 2.20)
Worse		0.86 (0.49, 1.42)	0.85 (0.49, 1.41)	0.84 (0.47, 1.38)	0.84 (0.47, 1.39)	0.87 (0.49, 1.44)
Out-group discrimination						
No difference		1.00	1.00	1.00	1.00	1.00
Better		1.10 (0.75, 1.61)	1.00 (0.68, 1.46)	0.98 (0.66, 1.42)	1.03 (0.69, 1.50)	1.02 (0.68, 1.48)
Worse		0.68 (0.36, 1.17)	0.77 (0.41, 1.34)	0.74 (0.39, 1.29)	0.74 (0.39, 1.29)	0.72 (0.38, 1.25)
Coronary heart disease (CHD)						
In-group discrimination						
No difference		1.00	1.00	1.00	1.00	1.00
Better		1.24 (0.80, 1.85)	1.16 (0.75, 1.73)	1.15 (0.74, 1.71)	1.16 (0.74, 1.73)	1.23 (0.79, 1.84)
Worse		0.83 (0.50, 1.29)	0.83 (0.50, 1.30)	0.82 (0.50, 1.29)	0.80 (0.48, 1.26)	0.84 (0.51, 1.33)
Out-group discrimination						
No difference		1.00	1.00	1.00	1.00	1.00
Better		1.37 (0.97, 1.93)	1.26 (0.89, 1.77)	1.22 (0.86, 1.72)	1.22 (0.86, 1.72)	1.23 (0.87, 1.73)
Worse		1.41 (0.92, 2.12)	1.54 (0.99, 2.31)	1.47 (0.95, 2.21)	1.44 (0.93, 2.18)	1.43 (0.92, 2.16)
Heart failure (HF)						
In-group discrimination						
No difference		1.00	1.00	1.00	1.00	1.00
Better		1.32 (0.93, 1.83)	1.24 (0.87, 1.72)	1.14 (0.80, 1.59)	1.17 (0.82, 1.63)	1.24 (0.87, 1.73)
Worse		0.97 (0.66, 1.37)	0.94 (0.64, 1.34)	0.87 (0.59, 1.24)	0.85 (0.58, 1.22)	0.89 (0.61, 1.27)
Out-group discrimination						
No difference		1.00	1.00	1.00	1.00	1.00
Better		1.05 (0.78, 1.39)	0.94 (0.70, 1.24)	0.91 (0.68, 1.20)	0.88 (0.66, 1.17)	0.89 (0.66, 1.18)
Worse		0.90 (0.61, 1.30)	1.13 (0.76, 1.64)	1.09 (0.73, 1.58)	1.04 (0.69, 1.51)	1.03 (0.69, 1.50)

Abbreviations: *HR* hazards ratio, *CI* confidence interval

Cardiovascular disease: *n* = 282; censored: *n* = 3237; total *N* = 3519

Stroke: *n* = 136; censored: *n* = 3383; total* N* = 3519

Coronary heart disease: *n* = 172; censored: *n* = 3347; total *N* = 3519

Heart failure: *n* = 258; censored: *n* = 3261; total *N* = 3519

^*^Cardiovascular disease: based on stroke and coronary heart disease variables

^†^Model 1: unadjusted

^‡^Model 2: Adjusted for age, sex

^§^Model 3: Adjusted for model 2 + education, income, occupation

||Model 4: Adjusted for model 3 + alcohol intake, smoking status, physical activity, diet, body mass index

^#^Model 5: Adjusted for model 4 + hypertension status and diabetes status

Out-group skin tone discrimination was not associated with an increased risk of CVD overall. The HR for individuals reporting better treatment compared to the no difference group was 1.16 [95% CI 0.88–1.51] and the HR for those reporting worse treatment was 1.02 (95% CI 0.69–1.45) suggesting there were no significant differences in the risk of overall CVD (Table 3, model 5).

### Stroke

The effect size for in-group skin tone discrimination indicated a higher risk of stroke for individuals reporting better treatment compared to no difference, but confidence intervals were wide (HR 1.41 [95% CI 0.87–2.20]) (Table 3, model 5). There were no significant differences in the risk of stroke for those reporting worse treatment compared to the no difference group (HR 0.87 [95% CI 0.49–1.44) (Table 3, model 5).

Out-group skin tone discrimination was not associated with an increased risk of stroke. The HR for individuals reporting better treatment versus the no difference group was 1.02 [95% CI 0.68–1.48], while the HR of those reporting worse treatment was 0.72 [95% CI 0.38–1.25], indicating no significant differences in the risk of stroke (Table 3, model 5).

### Coronary Heart Disease

The effect size for in-group skin tone discrimination indicated a higher risk of CHD for individuals reporting better treatment compared to no difference, but confidence intervals were wide (HR 1.23, 95% CI 0.79–1.84) (Table 3, model 5). No differences in CHD risk were observed for those reporting worse treatment compared to the no difference group (HR 0.84 [95% CI 0.51–1.33]) (Table 3, model 5).

Out-group skin tone discrimination was also marginally associated with incident CHD. The effect sizes indicated a higher risk of CHD for those who reported better treatment (HR 1.23 [95% CI 0.87–1.73]) or worse treatment (HR 1.43 [95% CI 0.92–2.16]) compared to those who reported no difference, but confidence intervals were wide (Table 3, model 5).

### Heart Failure

The effect size for in-group skin tone discrimination indicated a higher risk of HF for individuals reporting better treatment compared to no difference, but confidence intervals were wide (HR 1.24 [95% CI 0.87–1.73]) (Table 3, model 5). There were no significant differences in the risk of developing HF for those reporting worse treatment compared to the no difference group (HR 0.89 [95% CI 0.61–1.27]) (Table 3, model 5).

Out-group skin tone discrimination was not associated with an increased risk of HF. No significant differences in HF risk were observed for those reporting better treatment (HR 0.89 [95% CI 0.66–1.18] or worse treatment (HR 1.03 [95% CI 0.69–1.50] compared to the no difference group (Table 3, model 5).

### Interactions Between Skin Tone Discrimination and Sex

Sex did not moderate the associations between in-group skin tone discrimination and incident CVD overall (*p*-value for interaction = 0.37), stroke (*p*-value for interaction = 0.20), CHD (*p*-value for interaction = 0.27), and HF (*p*-value for interaction = 0.94) after full adjustment. Sex did not moderate the associations between out-group skin tone discrimination and incident CVD overall (*p*-value for interaction = 0.14), stroke (*p*-value for interaction = 0.18), CHD (*p*-value for interaction = 0.48), and HF (*p*-value for interaction = 0.95) when fully adjusted.

### Interactions Between Skin Tone Discrimination and Optimism

Optimism did not moderate the associations between in-group skin tone discrimination and incident CVD overall (*p*-value for interaction = 0.31), stroke (*p*-value for interaction = 0.13), CHD (*p*-value for interaction = 0.88), and HF (*p*-value for interaction = 0.48) in fully adjusted models. There was interaction between out-group skin tone discrimination and optimism for HF (*p*-value for interaction = 0.09) after full adjustment. Individuals who reported worse treatment and had the highest level of optimism, compared to average and low levels, had the greatest risk of HF (Table 4). Interaction between out-group skin tone discrimination and optimism was not observed for CVD overall (*p*-value = 0.17), stroke (*p*-value for interaction = 0.11), or CHD (*p*-value for interaction = 0.11).

**Table 4:** Associations between skin tone discrimination and incident heart failure stratified by optimism

	Heart failure		
	Low HR (95% CI)	Medium HR (95% CI)	High HR (95% CI)
In-group discrimination			
No difference	1.00 (ref)	1.00 (ref)	1.00 (ref)
Better	1.51 (0.93, 2.35)	1.13 (0.75, 1.63)	0.85 (0.39, 1.68)
Worse	0.93 (0.53, 1.55)	0.89 (0.59, 1.28)	0.84 (0.41, 1.59)
Out-group discrimination			
No difference	1.00 (ref)	1.00 (ref)	1.00 (ref)
Better	1.01 (0.66, 1.50)	0.86 (0.63, 1.16)	0.74 (0.42, 1.25)
Worse	0.71 (0.38, 1.24)	1.11 (0.73, 1.62)	1.73 (0.86, 3.22)

Abbreviations: *HR* hazards ratio, *CI* confidence interval

Adjusted for age, sex, education, income, occupation, alcohol intake, smoking status, physical activity, diet, body mass index, hypertension status, and diabetes status

### Sensitivity Analyses

Consistent with findings from the main analyses, time-varying in-group skin tone discrimination (and not out-group discrimination) was associated with a higher incidence of overall CVD (HR_better vs. no difference_ 1.44 [95% CI 1.04–1.95]) (Table S1).

Time-varying in-group skin tone discrimination (better treatment) was associated with incident stroke, CHD, and HF, but confidence intervals were wide. Time-varying out-group skin tone discrimination was not associated with any CVD outcomes (Table S1). There were no significant interactions between time-varying skin tone discrimination (in-group and out-group) and sex or optimism for any of the CVD outcomes (all *p*-values for interaction > 0.10).

## Discussion

Skin tone discrimination has been hypothesized to contribute to CVD, but previous literature on skin tone discrimination and CVD has been mostly cross-sectional, and even fewer studies examined whether these associations varied by sex and levels of optimism. The present study is the first to examine the impact of skin tone discrimination on incident CVD and whether sex and optimism moderated the relationship among a large African American adult population in the JHS.

### Skin Tone Discrimination and Incident CVD

Although the present study suggests that in-group discrimination was marginally associated with a higher incidence of CVD, this relationship was unexpectedly observed among those that perceived better treatment than other African American individuals due to their skin tone. Participants who reported that Black individuals treated them better than other Black individuals due to their skin tone had a higher risk of developing CVD compared to those that reported no difference in treatment based on skin tone. These findings are consistent with previous studies that reported an association between in-group skin tone discrimination and worse cardiovascular and cardiometabolic health [18], as well as self-rated physical health[10]; however, the previous studies asked about overall skin tone discrimination, instead of skin tone discrimination in relation to other Black individuals. It is possible that while African American individuals in the JHS experienced better treatment than other African American individuals, witnessing the mistreatment of their African American counterparts could initiate stress responses that could be detrimental to their cardiovascular health[41]. In support of this hypothesis, previous studies observed associations between vicarious discrimination (i.e., indirect experience of discrimination through witnessing or hearing about discriminatory acts against others[41]) and CVD risk[42, 43]. Participants may also experience heightened social stress associated with better treatment within their racial group. In addition, those who reported better treatment in the current study might have had a lighter skin tone, in which prior research found that individuals with lighter skin reported fewer experiences of discrimination compared to their counterparts with darker skin[44].

Out-group skin tone discrimination was marginally associated with incident CHD. Reporting better or worse treatment from White individuals resulted in a higher risk of CHD compared to those reporting no difference, suggesting that regardless of the perceived quality of treatment, any form of discrimination may be detrimental to health.

Our findings differ from previous studies that have reported no associations between out-group skin tone discrimination. For example, one study of 3415 native-born US Black individuals found that out-group skin tone discrimination was not associated with cardiovascular and cardiometabolic disorders[18]. Similarly, another study reported that out-group skin tone discrimination was not associated with hypertension and self-rated physical health for native-born US Black individuals[10]. The differences in findings could be attributed to the measures used. The current study assessed the degree of skin tone discrimination participants encountered relative to other Black individuals, whereas the previous studies asked participants how often they perceived discrimination due to their skin color from White individuals in general rather than relative to other Black people. Nonetheless, as expected, our findings align with broader research showing that discrimination negatively impacts cardiovascular health[7].

Unexpectedly, there was a general trend where participants who reported worse in-group treatment had a lower risk for the CVD outcomes. It is possible that these individuals may have anticipated mistreatment because of their skin tone, prompting them to develop proactive coping techniques, which may have effectively reduced stress arising from skin tone discrimination, lowering their risk of CVD[45, 46]. There was a similar trend for those reporting worse out-group treatment with stroke.

### Sex as a Moderator

Sex did not moderate the associations between in-group or out-group skin tone discrimination and any of the CVD health outcomes. These findings were consistent with one prior study that reported no sex differences in the relationship between skin tone discrimination and cardiovascular health[18]. Generally, skin tone discrimination has been posited to be gendered, with skin color playing a more significant role in the experiences of African American women than men[17]. However, the lack of substantial sex differences may imply that the impact of skin tone discrimination on health is just as important for African American men as it is for African American women[10]. The physiological responses to skin tone discrimination, such as increased stress levels, may not be inherently sex-specific[47].

### Optimism as a Moderator

Optimism moderated the association between out-group skin tone discrimination and incident HF. Individuals who reported worse treatment and reported the highest level of optimism had the greatest risk of HF. These findings were in contrast to another study where individuals with high versus low optimism had a lower risk of incident heart failure[48]. Individuals reporting higher levels of optimism may be more likely to downplay or dismiss their experiences of discrimination. This denial may prevent individuals from addressing the underlying stressors from discrimination that contribute to poor health over time as they downplay symptoms, delay seeking medical services, or fail to take necessary preventative measures because they feel their positive outlook will naturally overcome the health problems from discrimination[49].

### Limitations

Limitations need to be considered when interpreting the findings from the present study. First, these findings may not be generalizable to African American individuals in other geographical locations since the study population was comprised of only African American adults from Jackson, Mississippi. Additionally, there were multiple limitations with the skin tone discrimination measures. First, the skin tone discrimination measures were based on unfair treatment relative to other Black individuals, and comparisons were not made with other racial and/or ethnic groups. Consequently, this measure may not account for or accurately capture skin tone discrimination experiences. Furthermore, the measure did not specify how participants were mistreated, which is important, as different forms of mistreatment—such as microaggressions versus overt discrimination[50]—may have varying effects on health. Similarly, the measure did not identify where the discrimination occurred. The setting in which discrimination takes place may influence both the frequency [51] and impact of mistreatment. Another limitation is that the skin tone discrimination measure did not account for participants’ shade of skin (e.g., lighter skin or darker skin), which may not capture the full dimension of skin tone discrimination. Additionally, the measure of in-group skin tone discrimination did not include the skin tone of the Black individuals engaging in the treatment and could not capture whether discrimination was more frequently perpetuated by individuals with lighter skin or darker skin. This measure therefore fails to capture important hierarchical dynamics and the full complexity of skin tone discrimination. Lastly, another limitation is the wide confidence intervals, which indicate a lack of precision in the estimates, possibly resulting from small sample sizes, sparse data, and high variability in the data.

### Strengths

Despite these limitations, this study has several strengths. This study was performed using a large cohort of African American adults. The prospective study design allowed for establishing temporality and making causal inferences about the associations between skin tone discrimination and CVD. In addition, the data allowed for the assessment of time-varying skin tone discrimination in sensitivity analyses, which was important given that skin tone discrimination may be context and time dependent [35, 36]. Furthermore, in recognizing the potential adverse effects of discrimination on health, this study addresses knowledge gaps by exploring optimism as a moderator of the relationship between skin tone discrimination and CVD. Lastly, this study used multiple robust, verified objective measures of CVD events rather than self-reported measures that may be subject to recall bias [52].

### Future Research

Future research is needed to confirm the findings on skin tone discrimination and cardiovascular outcomes that disproportionately impact African American adults. Future studies could also include additional measures of skin tone discrimination (e.g., using self-rated skin tone, interviewer rated skin tone, and spectrometer-based skin tone) to accurately capture skin tone discrimination. Studies could also explore skin tone discrimination and CVD incidence in other Black heritage groups where skin tone discrimination is pervasive, including Afro-Latino and Afro-Caribbean populations. Future research could also explore regional differences in the association between skin tone discrimination and CVD across the USA, since the current study was conducted in one state in the southern region of the USA.

Although this study adjusted for several sociodemographic characteristics and CVD risk factors, future studies might consider other potential mediators (e.g., biomarkers of chronic stress, inflammation access to health care) or moderators (e.g., coping mechanisms, resilience strategies). Chronic stress is a well-documented risk factor for CVD[53], whereby prolonged exposure to stress has been linked to physiological dysregulation and increased disease risk[54, 55] For example, one study found that C-reactive protein (CRP)—a marker of inflammation and stress—mediated the relationship between perceived discrimination and cardiovascular conditions, including CVD, hypertension, and stroke/transient ischemic attack, suggesting that stress may be a biological pathway linking discrimination to a greater burden of cardiovascular disease[56]. Additionally, coping strategies may play a significant role in shaping cardiovascular outcomes. One study found that religious coping buffered the negative effect of discrimination on CVD for African American men, but not women [57]. Similarly, another study reported that African American individuals who experienced a high discrimination burden with greater high-effort coping scores were more likely to be a part of an inflammatory risk profile characterized by elevated CRP and urinary albumin—both of which are associated with increased cardiovascular risk[58]. Furthermore, studies have shown that active coping[59] and John Henryism[60] exacerbated the relationship between discrimination and hypertension, a major risk factor for CVD for African American individuals. Lastly, future research could explore additional factors (e.g., self-efficacy) that may serve as protective factors in the relationship between skin tone discrimination and CVD.

## Conclusions

Ultimately, the present study highlighted the differential impact of in-group and out-group skin tone discrimination on cardiovascular health. Better in-group treatment was marginally linked to a higher CVD risk, while out-group skin tone discrimination, whether better or worse treatment, marginally increased CHD risk. Skin tone discrimination may therefore be a unique social determinant of health separate from everyday and lifetime discrimination measures[18]. Including measures of skin tone discrimination will be essential to gaining a nuanced understanding of how discrimination contributes to the cardiovascular health disparities that disproportionately impact African American adults in the USA.

## Supplementary Information

Below is the link to the electronic supplementary material.

Supplementary file1 (PDF 264 KB)

## Data Availability

The JHS data and materials can be requested from the JHS Committee at https://www.jacksonheartstudy.org/Research/Study-Data/Data-Access.

## Declarations

### Disclaimer

This research was supported [in part] by the Intramural Research Program of the National Institutes of Health (NIH). The contributions of the NIH author(s) were made as part of their official duties as NIH federal employees, are in compliance with agency policy requirements, and are considered Works of the United States Government. However, the findings and conclusions presented in this paper are those of the author(s) and do not necessarily reflect the views of the NIH or the U.S. Department of Health and Human Services.
