# Supplementary material for: Experiences of In-Group and Out-Group Skin Tone Discrimination and Their Associations with Incident Cardiovascular Disease Among African American Adults in the Jackson Heart Study
Source: J Racial Ethn Health Disparities. 2025 Aug 7;13(2):1594–607. doi: 10.1007/s40615-025-02590-8 (PMC12966262; doi:10.1007/s40615-025-02590-8)
Supplement: Supplementary file 1 — Supplementary file1 (PDF 264 KB) [file 40615_2025_2590_MOESM1_ESM.pdf]

Experiences of In-group and Out-group Skin Tone Discrimination and their Associations with Incident Cardiovascular Disease among African American Adults in the Jackson Heart Study

Sydney A. Barlow B.S; Jessica R. Fernandez, PhD; Juliana S. Sherchan B.S; Ellis P. Monk, PhD; Jaime Slaughter-Acey, PhD, MPH; Mario Sims, PhD, MS, Allana T. Forde, PhD, MPH

Supplemental Material

Table S1: Associations between Time-varying Skin Tone Discrimination and Incident Cardiovascular Disease

		HR (95 % CI)				
		Model 1†	Model 2‡	Model 3§	Model 4‖	Model 5#
**Cardiovascular Disease***						
In-group discrimination						
	No difference	1.00	1.00	1.00	1.00	1.00
	Better	1.47 (1.06, 1.98)	1.42 (1.03 1.92)	1.38 (1.00, 1.87)	1.39 (1.01, 1.89)	1.44 (1.04, 1.95)
	Worse	0.85 (0.58, 1.21)	0.88 (0.60, 1.25)	0.87 (0.59, 1.24)	0.86 (0.58, 1.22)	0.88 (0.60, 1.25)
Out-group discrimination						
	No difference	1.00	1.00	1.00	1.00	1.00
	Better	1.16 (0.88, 1.50)	1.09 (0.83 1.42)	1.06 (0.81, 1.38)	1.08 (0.82, 1.41)	1.07 (0.82 1.40)
	Worse	0.74 (0.48, 1.08)	0.84 (0.55, 1.24)	0.81 (0.53, 1.20)	0.81 (0.52, 1.19)	0.81 (0.53, 1.20)
**Stroke**						
In-group discrimination						
	No difference	1.00	1.00	1.00	1.00	1.00
	Better	1.53 (0.97, 2.34)	1.50 (0.95, 2.29)	1.45 (0.91, 2.22)	1.47 (0.92, 2.27)	1.51 (0.94, 2.32)
	Worse	0.87 (0.49, 1.43)	0.90 (0.51, 1.49)	0.89 (0.51, 1.48)	0.89 (0.50, 1.46)	0.90 (0.51. 1.49)
Out-group discrimination						
	No difference	1.00	1.00	1.00	1.00	1.00
	Better	0.93 (0.62, 1.37)	0.87 (0.58, 1.28)	0.86 (0.57, 1.26)	0.90 (0.60, 1.33)	0.89 (0.59, 1.32)
	Worse	0.52 (0.25, 0.95)	0.62 (0.30, 1.14)	0.60 (0.29, 1.11)	0.61 (0.30, 1.12)	0.61 (0.30, 1.12)
**Coronary Heart Disease (CHD)**						
In-group discrimination						
	No difference	1.00	1.00	1.00	1.00	1.00
	Better	1.34 (0.88, 1.98)	1.29 (0.85, 1.91)	1.27 (0.83, 1.88)	1.28 (0.84, 1.90)	1.32 (0.86, 1.96)
	Worse	0.74 (0.44, 1.18)	0.77 (0.46, 1.23)	0.76 (0.45, 1.22)	0.75 (0.44, 1.19)	0.77 (0.45, 1.24)
Out-group discrimination						
	No difference	1.00	1.00	1.00	1.00	1.00
	Better	1.28 (0.91, 1.78)	1.21 (0.86, 1.68)	1.17 (0.83, 1.63)	1.17 (0.83, 1.64)	1.17 (0.83, 1.64)
	Worse	0.95 (0.57, 1.50)	1.06 (0.64, 1.69)	1.03 (0.61, 1.63)	1.01 (0.61, 1.61)	1.03 (0.61, 1.64)
**Heart Failure (HF)**						
In-group discrimination						
	No difference	1.00	1.00	1.00	1.00	1.00
	Better	1.35 (0.96, 1.87)	1.31 (0.93, 1.81)	1.24 (0.88, 1.72)	1.28 (0.91, 1.78)	1.33 (0.94, 1.84)
	Worse	0.88 (0.59, 1.26)	0.89 (0.59, 1.28)	0.83 (0.56, 1.20)	0.83 (0.55, 1.19)	0.85 (0.57, 1.23)
Out-group discrimination						
	No difference	1.00	1.00	1.00	1.00	1.00
	Better	1.11 (0.83, 1.46)	1.01 (0.76, 1.34)	0.99 (0.74, 1.31)	0.97 (0.73, 1.29)	0.97 (0.73, 1.28)
	Worse	0.85 (0.56, 1.25)	1.11 (0.73, 1.63)	1.10 (0.72, 1.62)	1.07 (0.70, 1.58)	1.09 (0.71, 1.60)

Abbreviations: HR, Hazards ratio; CI, confidence interval.
Cardiovascular Disease: n = 282; Censored: n = 3237; Total N = 3519
Stroke: n = 136; Censored: n = 3383; Total N = 3519
Coronary Heart Disease: n = 172; Censored: n = 3347; Total N = 3519
Heart Failure: n = 258; Censored: n = 3261; Total N = 3519
*Cardiovascular disease: Based on stroke and coronary heart disease variables
†Model 1: unadjusted
‡Model 2: Adjusted for age, sex
§Model 3: Adjusted for model 2 + education, income, occupation
||Model 4: Adjusted for model 3 + alcohol intake, smoking status, physical activity, diet, body mass index
#Model 5: Adjusted for model 4 + hypertension status and diabetes status
